# Catalase-Like Antioxidant Activity is Unaltered in Hypochlorous Acid Oxidized Horse Heart Myoglobin

**DOI:** 10.3390/antiox8090414

**Published:** 2019-09-18

**Authors:** Gulfam Ahmad, Belal Chami, Mary El Kazzi, Xiaosuo Wang, Maria Tereza S. Moreira, Natasha Hamilton, Aung Min Maw, Thomas W. Hambly, Paul K. Witting

**Affiliations:** 1 Redox Biology Group, Discipline of Pathology, Faculty of Medicine and Health, Charles Perkins Centre, The University of Sydney, Sydney, NSW 2006, Australia; gulfam.ahmad@sydney.edu.au (G.A.); mael2199@uni.sydney.edu.au (M.E.K.); xiaosuo.wang@sydney.edu.au (X.W.); mariatereza.sm@gmail.com (M.T.S.M.); natasha.hamilton@sydney.edu.au (N.H.); amaw2321@uni.sydney.edu.au (A.M.M.); t.hambly@gmail.com (T.W.H.); 2 Discipline of Oral Pathology, Faculty of Medicine and Health, Charles Perkins Centre, The University of Sydney, Sydney, NSW 2006, Australia; belal.chami@sydney.edu.au

**Keywords:** myoglobin, hypochlorous acid, protein oxidation, haem-iron reduction, MetMb reductase, catalase-like antioxidant activity

## Abstract

Activated neutrophils release myeloperoxidase that produces the potent oxidant hypochlorous acid (HOCl). Exposure of the oxygen transport protein horse heart myoglobin (hhMb) to HOCl inhibits Iron III (Fe(III))-heme reduction by cytochrome *b*5 to oxygen-binding Iron II (Fe(II))Mb. Pathological concentrations of HOCl yielded myoglobin oxidation products of increased electrophoretic mobility and markedly different UV/Vis absorbance. Mass analysis indicated HOCl caused successive mass increases of 16 a.m.u., consistent serial addition of molecular oxygen to the protein. By contrast, parallel analysis of protein chlorination by quantitative mass spectrometry revealed a comparatively minor increase in the 3-chlorotyrosine/tyrosine ratio. Pre-treatment of hhMb with HOCl affected the peroxidase reaction between the hemoprotein and H_2_O_2_ as judged by a HOCl dose-dependent decrease in spin-trapped tyrosyl radical detected by electron paramagnetic resonance (EPR) spectroscopy and the rate constant of 2,2′-azino-bis(3-ethylbenzothiazoline-6-sulphonic acid (ABTS) oxidation. By contrast, Mb catalase-like antioxidant activity remained unchanged under the same conditions. Notably, HOCl-modification of Mb decreased the rate of ferric-to-ferrous Mb reduction by a cytochrome b5 reductase system. Taken together, these data indicate oxidizing HOCl promotes Mb oxidation but not chlorination and that oxidized Mb shows altered Mb peroxidase-like activity and diminished rates of one-electron reduction by cytochrome b5 reductase, possibly affecting oxygen storage and transport however, Mb-catalase-like antioxidant activity remains unchanged.

## 1. Introduction

Neutrophils are recruited to heart tissues after acute myocardial infarction (AMI); recruitment occurs within the first 24 h as part of the early innate inflammatory response post AMI [1,2]. Activated neutrophils release myeloperoxidase (MPO) that produces the potent oxidant hypochlorous acid (HOCl), which is cell permeable and known to cause oxidative modification of proteins and low-molecular weight biomolecules [3,4]. In the case of proteins, oxidizing HOCl reacts most rapidly with sulfur containing residues such as cysteine and methionine, followed by protein-side chains containing lysine, histidine, tryptophan and tyrosine [5,6]. Intact proteins become oxidized at these reactive amino centers to yield oxidized and/or chlorinated products (e.g., for tyrosine dimeric, hydroxylated or chlorinated products can be formed largely dependent on the oxidant involved).

The oxygen transport protein myoglobin (Mb) is a predominant protein in skeletal and cardiac muscle [7]. Therefore, Mb is potentially a primary target for oxidizing HOCl produced by neutrophils recruited to the myocardium following a heart attack, potentially leading to a secondary oxidative process that may further inhibit cardiac function post-AMI [8]. The primary function of Mb in muscle cells is generally considered as an oxygen transport/storage protein [9] although its role in the maintenance and regulation of redox active molecules such as hydrogen peroxide (H_2_O_2_) and nitric oxide (NO) are other important functions for this protein. In the case of NO, Mb can diminish the concentration of this vasoactive molecule through oxidation [10,11] and/or increase NO concentrating through Mb’s inherent nitrite reductase activity [12]. In particular, the ability of Mb to act as a nitrite reductase has been linked to regulation of muscle contractile function [13]. Furthermore, cardiac Mb is also a pseudo-peroxidase that also exhibits a catalase-like antioxidant activity [14]; both enzymic activities may be important in regulating levels of cardiac hydrogen peroxide (H_2_O_2_) particularly following acute ischemia-reperfusion injury post AMI.

Here we identify that Mb oxidation by reagent HOCl decreases the efficiency of the one-electron reduction between ferric Mb [MbFe(III)] by a cytochrome *b*5 reductase system (considered to represent the intracellular Mb reductase necessary to maintain the ferrous Mb (MbFe(II)) form. Changes to the maintenance of intracellular MbFe(II) potentially impacts on oxygen-binding/transport to mitochondria for oxidative phosphorylation and cellular energetics.

## 2. Material and Methods

### 2.1. Materials

Purified cytochrome *b*5 and human NADPH-P450 reductase were purchased from Invitrogen (Sydney, Australia). These two proteins are central to the intracellular Mb-reductase system [15]. All buffers were prepared using Nanopure filtered water followed by storage over Chelex-100^®^ resin (BioRad, Sydney, Australia) at 4 °C for at least 24 h to remove contaminating transition metal ions. Solutions of stock HOCl (Merck, Sydney, Australia) were standardized by using A_292nm_ (^−^OCl) = 350 M^−1^ cm^−1^ and A_235nm_ (HOCl) = 100 M^−1^ cm^−1^ [16]. The stock was diluted into phosphate buffered saline (PBS, 250 mM, pH 7.4) prior to use. Horse heart Mb (hhMb) was obtained from Sigma Aldrich (Sydney, Australia) and stock solutions of this protein were prepared immediately before use in 250 mM PBS (pH 7.4) and standardized using _409_ = 188,500 M^−1^ cm^−1^ [17]. This concentration of phosphate buffer was selected to avoid alterations in pH upon addition of reagent HOCl as increased pH may interfere with the reaction between the oxidant and hhMb. Unless specified otherwise, all other chemicals employed were analytical grade reagents.

### 2.2. Experimental Section

#### 2.2.1. Oxidation Reactions

All oxidation reactions were carried out at 37 °C in 250 mM PBS (pH 7.4) containing hhMb at the final concentrations indicated in the legends to the figures. The buffer concentration employed here was used to decrease the possibility that added HOCl altered the pH of the reaction mixture upon addition to hhMb, which may affect the tertiary structure and hence availability of amino acids to interact with the oxidizing reagent HOCl. All reagents and protein solutions were freshly prepared, and reactions were initiated by addition of reagent HOCl (ranging 0−50 mol/mol excess of the oxidant relative to hhMb) or water (as vehicle control) to the protein with rapid mixing. This in vitro system was designed to model the reaction of cardiac Mb in the presence of activated leukocytes that release extracellular MPO, the enzyme responsible for generating oxidizing HOCl. Oxidized proteins were prepared immediately prior to use in the designated analytical studies. Final HOCl/hhMb (mol/mol) ratios were as specified in the legends to the figures. A dose range of HOCl/hhMb ratios was tested here since the precise concentration of HOCl produced by leukocytes recruited to an area of inflammation is not clear. Whether the dose range employed is pathophysiologically relevant in vivo is somewhat dependent on the level of leukocyte recruitment to an inflammatory region of tissue, the degree of cellular activation to promote degranulation and activation of extracellular MPO and the prevalence of biological targets in the local region of the organ investigated (i.e., here we targeted Mb that is present at a relatively high concentration in myocardial muscle).

#### 2.2.2. Electrophoretic Mobility of Native and HOCl-Modified hhMb

The electrophoretic mobility of native and HOCl-modified hhMb was determined by using a protocol originally established to monitor oxidation status of low-density lipoprotein [18]. Briefly, electrophoresis was performed with an agarose gel (0.8% agarose in TAE buffer) and staining with Coomassie Brilliant Blue R-250 to highlight protein migration. Electrophoresis was performed at 100 V over a period of 30 min and the relative electrophoretic mobility (REM) was defined as the relative distance migrated from the origin by HOCl-oxidized hhMb compared to the native hhMb in the absence of HOCl (control) with the cathode (−) placed at the top of the gel closest to the loading comb and the anode (+) at the bottom.

#### 2.2.3. UV-Vis Absorbance Spectroscopy

Steady state visible spectra of native and oxidized hhMb were accumulated using a Beckman Coulter DU800 UV/Vis Spectrophotometer (Beckman Coulter Australia Pty Ltd, Sydney, Australia) maintained at 25 ± 0.1 °C with a Peltier temperature controller (Beckman Coulter Australia Pty Ltd, Sydney, Australia). To assess changes to baseline spectral characteristics, solutions of hhMb (ranging up to 50 µM) were treated with increasing doses of HOCl (final mole ratio ranging 0.5–50 mol/mol) and immediately transferred to a 1 mL quartz cuvette for spectral analysis in the range 500–600 nm). To conduct peroxidase activity measurements through the monitoring of rates of substrate oxidation in the presence of hhMb, a stock solution of 2,2′-azino-di-(3-ethyl)benzthiazoline-6-sulfonic acid (ABTS) was freshly prepared containing: 5 mM ABTS and 50 µM H_2_O_2_ in 0.1 M potassium phosphate buffer (pH 7.0). The substrate stock solution was placed into a 1 mL volume quartz cuvette (path length: 1 cm) and hhMb (final concentration 10 µM) was added to the mixture and the time-dependent accumulation of the ABTS radical cation was monitored at A_734 nm_ using a Beckman Coulter DU800 UV/Vis Spectrophotometer maintained at 25 ± 0.1 °C with an automated Peltier temperature controller. All pseudo first-order rate constants were determined as linear time dependent change in A_734 nm_ averaged over at least three independent rate determinations for each concentration of HOCl tested.

#### 2.2.4. Assessment of 3-Chlorotyrosine (3-Cl-Tyr)

The biomarker for HOCl oxidation of tyrosine (3-Cl-Tyr) was assessed by quantitative liquid chromatography with tandem mass spectrometry (LC–MS/MS) as described previously [19,20]. Briefly, 100 µg of (native or HOCl-modified) hhMb was precipitated in 3% *w*/*v* sodium deoxycholate and 50% *w*/*v* trichloroacetic acid/H_2_O, followed by centrifugation (6000× *g*, 5 min) to pellet the solid protein. Protein pellets were washed in ice-cold acetone (2 × 0.5 mL) and then dried under a stream of N_2(g)_ before adding 6 M methane sulfonic acid. The acidified mixture was finally hydrolyzed under vacuum (110 °C) and after ~18 h, the dark hydrolysate was resuspended in Nanopure water and purified using solid phase extraction cartridges (Superclean Envi-Chrom, 250 mg, Sigma-Aldrich, Sydney, Australia). Detection and quantification of 3-Cl-Tyr, unmodified tyrosine and corresponding labeled internal standards in these purified hydrolysates was conducted using an Agilent 6460 triple quad mass spectrometer (Agilent Technologies Australia Pty Ltd, Mulgrave Victoria, Australia) coupled with 1290 LC series (Agilent Technologies Australia Pty Ltd, Mulgrave Victoria, Australia) as described previously [20].

#### 2.2.5. Liquid Chromatography and Electrospray Mass Spectrometry

Final reaction mixtures were diluted in MilliQ Water to yield ~1 µM protein and then aspirated and desalted using a ZipTip cartridge (Merck-Millipore, Sydney Australia) to bind, wash and elute the purified protein. Purified proteins were then subjected to two procedures as outlined below:

1. Proteins were separated at a flow rate of 0.4 mL/min on a C18 reverse-phase column (particle size 3 μm, 3 mm × 150 mm) using solvent A (0.1% *v*/*v* trifluoroacetic acid in water) and solvent B (0.1% *v*/*v* trifluoroacetic acid in CH_3_CN) and products were detected by absorbance at 210 nm to assess the impact of HOCl-oxidation on hhMb (as measured by changes in retention time for modified protein(s) eluting from the column).
2. Where required, mass analyses were performed on desalted protein samples (Ziptips Cat# Z720070); Merck-Millipore in positive ion mode with a Finnigan LCQ Deca XP ion trap instrument (Thermo Fisher Scientific, San Jose, CA, USA) coupled to a Finnigan Surveyor HPLC system (Thermo Fisher Scientific, San Jose, CA, USA) as described in detail elsewhere [21]. Modified hhMb proteins were injected directly to the electrospray MS under the following parameters: Electrospray needle was held at 4500 V; sheath gas was nitrogen set at 80 units; collision gas was helium and the temperature of the heated capillary was 250 °C. This analytical approach consistently resulted in detection of the apo-protein without the haem moiety as the haem group is non-covalently bound to the protein.

#### 2.2.6. Electron Paramagnetic Resonance and Spin Trapping Studies

Standard X-band electron paramagnetic resonance (EPR) spectra were obtained at 22 °C with a Bruker EMX Benchtop spectrometer (Bruker Pty Ltd, Preston, Victoria, Australia) as described previously [22]. Separate solutions of native or HOCl-oxidized hhMb (0.5 mM) were treated with H_2_O_2_ (ratio H_2_O_2_:hhMb ∼5 mol/mol) in the presence or absence of activated charcoal-purified spin trap [23] 5,5-dimethyl-1-pyrroline N-oxide (DMPO; Sigma-Aldrich, Sydney Australia; final concentration 5 mM). Next, a sample of the reaction mixture (250 μL) was removed and rapidly transferred into a standard quartz flat cell (Wilmad, Buena, NJ, USA) for EPR analyses at 22 °C. The limit of detection of a stable nitroxide (TEMPO) measured under identical conditions was determined to be ∼50 nM. EPR spectra were obtained as an average of five cumulative scans with a modulation frequency of 100 kHz and a sweep time of 84 s. Microwave power and modulation amplitude used for each analysis varied appropriately as indicated in the figure legends. The metal chelator diethylenetriaminepentaacetic acid (DTPA, final concentration 100 μM) was included in reaction mixtures to minimize the possibility of peroxide decomposition by Fenton-type chemistry.

#### 2.2.7. Mb Catalase-Like Activity

Assessment of hhMb catalase activity was determined monitoring the reaction between hhMb and two peroxide sources; H_2_O_2_ (30% *v*/*v*) or cumene hydroperoxide (99.6% purity) were from Sigma-Aldrich (Sydney, Australia). Working solutions of peroxide (final concentration 200 μM) were mixed with native or HOCl-modified hhMb (final concentration) loss of peroxide was examined after 15 min reaction at 22 °C, where the residual levels of peroxide were determined using a modified Ferrous *Oxidation*−Xylenol Orange (FOX)-1 assay as described in detail elsewhere [24]. Briefly, an aliquot of (250 µM ammonium sulfate, 100 mM D-sorbitol and 125 µM xylenol orange) was then added to the reaction mixture of peroxide and Mb and incubated at 22 °C for 30 min. Finally, the colormetric reaction was read at 560 nm using a FLUOstar Omega plate reader (BMG LabTech, Mornington Victoria, Australia) and all quantitative data was normalized to the total protein (determined by standard bicinchoninic acid assay and using bovine serum albumin as the protein standard (Sigma-Aldrich, Sydney Australia).

#### 2.2.8. Reduction of Myoglobin by Cytochrome *b*5 Reductase System

Redox reactions with hhMb (50 μM final concentration) were performed at 22 °C in 250 mM PBS (pH 7.4) and initiated by adding HOCl (0, 5- and 10-fold mol excess). Reactions were terminated by adding 12.5 mM L-Met on ice to scavenge excess HOCl and then gel filtration (disposable PD 10 Desalting Columns; GE Healthcare, Sydney, Australia) to remove residual L-Met and any low-molecular weight oxidation products. Subsequent to protein oxidation and gel purification, the one-electron reduction of native and HOCl-oxidized ferric hhMb by added cytochrome *b*5 was investigated as described in detail elsewhere [25,26]. Briefly, a sample (10 μL) of the hhMb preparation (final Mb concentration 10 μM) was added to a reaction mixture containing 0.12 μM human recombinant cytochrome *b*5, 0.05 μM human NADPH-P450 reductase, glucose-6-phosphate (50 U), catalase (375 U), 20 mM glucose, 0.2 mM EDTA and 2 mM NADP^+^ in 100 mM phosphate buffer (pH 7.4). The reaction was carried out under air in a quartz cuvette and spectra (350 to 600 nm) recorded every 1 min against a reference sample containing the reaction mixture without Mb, using a Beckman Coulter DU800 UV/Vis Spectrophotometer maintained at 25 ± 0.1 °C. All pseudo first-order rate constants were determined as averages of at least three separate determinations.

#### 2.2.9. Statistical Analyses

All analyses were performed with Prism software (v3.0, GraphPad Inc., San Diego, CA, USA). Differences between groups were assessed with a one-way ANOVA and Tukey’s multiple comparison test to minimize type-1 and type-2 statistical error. A value of *p* < 0.05 was considered significant.

## 3. Results

**Figure 1:**
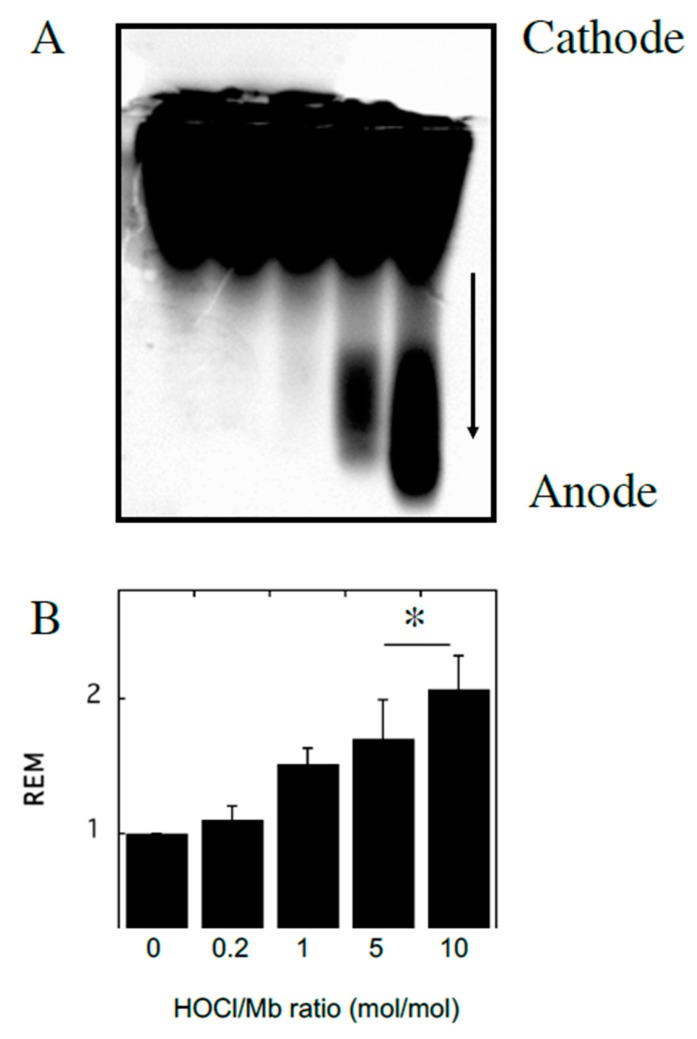
Modification of horse heart myoglobin (hhMb) with reagent HOCl increases electrophoretic mobility of the protein. Solutions of hhMb (final concentration 25 µM) were exposed to increasing dose of reagent HOCl (final ratio HOCl/hhMb mol/mol indicated). An aliquot was diluted 10× in phosphate buffer (50 mM, pH 7.5) and (**A**) loaded onto an agarose gel and protein separated by an applied electric field and stained using Coomassie Blue as described in the Methods section (arrow indicates direction of migration in the electric field). (**B**) Relative electrophoretic mobility (REM; measured as the distance travelled in the gel) was then calculated for each reaction condition and expressed as mean ± SD; *n* = 3. * Different to the vehicle control in the absence of added reagent HOCl; *p* < 0.05.

**Figure 2:**
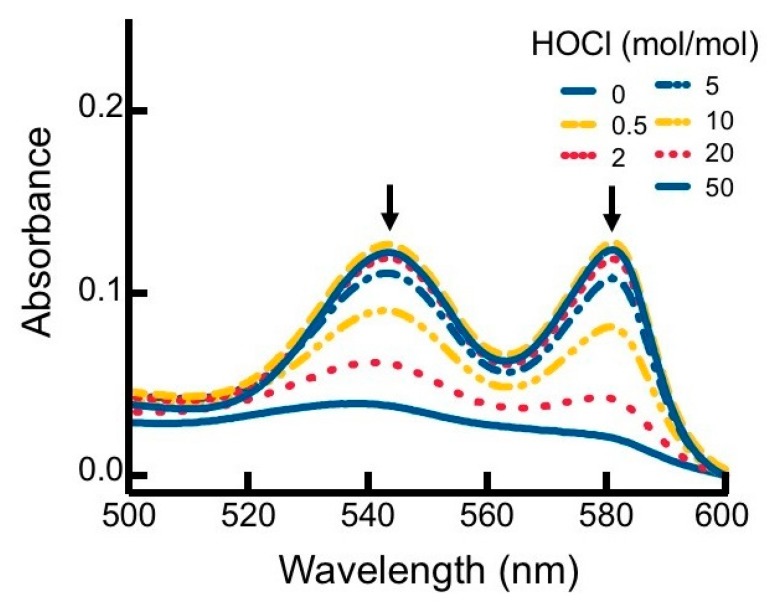
Alterations to the visible region of the hhMb absorbance spectrum in the presence of regent HOCl. Solutions of hhMb (final concentration 10 µM) were exposed to increasing dose of reagent HOCl (final ratio HOCl/hhMb mol/mol indicated). Aliquots of the reaction mixture were diluted (10×) in phosphate buffer (50 mM, pH 7.5) and transferred to a quartz cuvette and absorbance measured in the region 500–600 nm. Data are representative of *n* = 3 independent experiments using freshly prepared HOCl-modified hhMb. Arrows indicate a diminution in absorbance maxima at 542 and 580 nm.

To investigate the impact of reagent HOCl on myoglobin, native hhMb was treated with varying concentrations of reagent HOCl (Figure 1). A dose-dependent increase in post-translational modification to hhMb was observed as assessed by increased production of myoglobin oxidation products showing enhanced electrophoretic mobility (Figure 1; arrow denotes direction of in-gel migration with cathode and anode labeled) compared to vehicle control. Increased protein electrophoretic mobility was paralleled with marked changes to the visible region of the Mb electronic spectrum measured at pH 7.4 for HOCl-modified hhMb over a similar dose-range of this oxidant (Figure 2). Thus, HOCl stimulated a dose-dependent diminution in absorbance maxima observed at 542 and 580 nm (Figure 2). Taken together these findings indicate HOCl stimulated post-translational changes in native hhMb. This conclusion is supported by the altered electrophoretic mobility that manifested as HOCl-oxidation products of larger charge-to-size ratio migrating at a faster rate than the native protein towards the anode and HOCl-oxidation impacting on the active site binding of the haem moiety, which in-turn alters the electronic absorbance characteristics of ferric hhMb.

**Figure 3:**
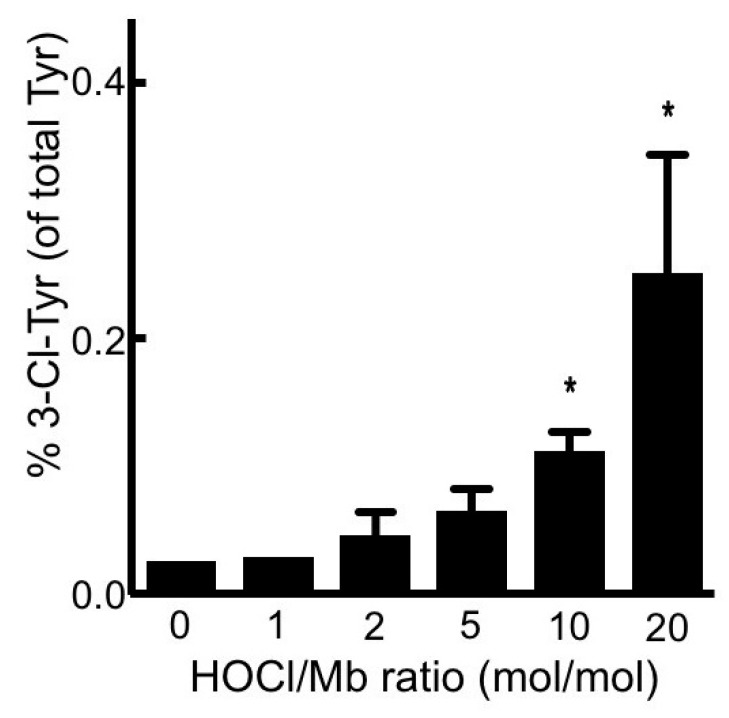
Treatment of hhMb with reagent HOCl increases the level of 3-chlorotyrosine tyrosine relative to native tyrosine in the protein. Solutions of hhMb (final concentration 50 µM) were exposed to increasing dose of reagent HOCl (final ratio HOCl/hhMb mol/mol indicated). Aliquots of reaction mixture were precipitated in ice-cold acetone (total 100 µg protein) and then processed and purified for quantitative mass spectrometry as described in the Methods section. Data represent mean ± SD; *n* = 3 independent preparations of hhMb. *Different to the vehicle control in the absence of added reagent HOCl; *p* < 0.05.

Neutrophil-HOCl is known to damage the proteins by chlorination of tyrosine forming 3-chlorotyrosine (a specific marker of HOCl protein damage) [27]. To confirm whether this form of damage occurred in hhMb exposed to reagent HOCl, levels of 3-chloro-tyrosine were measured and compared in native and HOCl-treated hhMb. Analysis of protein chlorination by quantitative mass spectrometry revealed an increase in the 3-chlorotyrosine/tyrosine ratio in Mb exposed to HOCl at ratios ≥ 10 mol/mol (Figure 3). The response to treatment with reagent HOCl was again dose-dependent when compared to vehicle control albeit the proportion of tyrosine modified by chlorination remained extremely low with only ~0.2 ± 0.1% of total tyrosine detected as the 3-Cl-Tyr modified product at the highest dose of reagent HOCl tested in our hands. This later observation indicated that the 3-Cl-Tyr modified product was formed in low overall yield and was unlikely to be causally linked to other physical-chemical changes in hhMb exposed to reagent HOCl.

**Figure 4:**
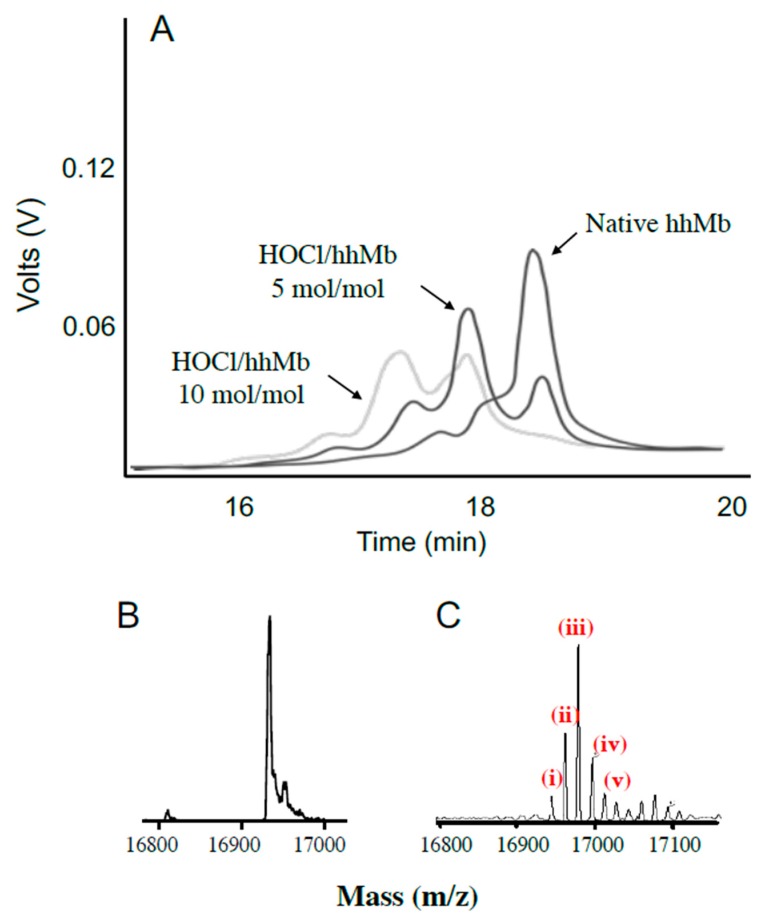
Oxidation of hhMb with reagent HOCl generates a complex mixture of products. Solutions of hhMb were exposed to vehicle (control) or 5 and 10 mol/mol excess reagent HOCl. Aliquots of control or HOCl-modified hhMb were then applied to a chromatographic system and the protein elution profile was monitored as described in the Methods section. Panel (**A**) shows several representative expanded elution profiles for hhMb eluting between 16–20 min. The deconvoluted mass spectrum of hhMb treated with vehicle control (seen primarily as a single peak of mass 16951.2 *m*/*z*. (Panel (**B**)) or 10 mol HOCl/mol hhMb protein (shown in Panel (**C**)) detected as multiple peaks identified by different masses ranging from ~16,966 to ~16,999 *m*/*z*. In panel C, the labeled peaks correspond to (i) residual native protein (16,951.2 *m*/*z*); mass peak at (ii) 16,966.9 (assigned as hhMb + 15.7 *m*/*z*. corresponding to ~1 oxygen atom), (iii) 16,983.2 (assigned as hhMb + 31.7 *m*/*z* corresponding to ~2 oxygen atoms), (iv) 16,999.0 (assigned as hhMb + 47.8 *m*/*z* corresponding to ~3 oxygen atoms) and (v) 17,015.3 (assigned as +63.8 *m*/*z* corresponding to ~4 oxygen atoms). These latter peaks corresponded to the sequential addition of 1–4 oxygen atoms to the protein backbone.

After establishing that low-level chlorination could occur in this system, we sought to confirm whether other post-translational amino acid modifications occurred after exposing hhMb to reagent HOCl (final ratios hhMb:HOCl ranging 0, 5 or 10 mol/mol). Mixtures of hhMb and HOCl were initially separated by liquid chromatography (Figure 4A). In the absence of HOCl, native Mb eluted primarily as a single major peak eluting at 18.5 min with minor shoulder peaks at ~18 min. Treating hhMb with 5 mol HOCl/mol protein markedly diminished the large peak eluting at 18.5 min (corresponding to native hhMb) and new products were detected eluting at 16.6, 17.5 and ~18 min. The intensity of these product peaks increased further upon exposing hhMb to 10 mol HOCl/mol protein where almost all native hhMb was depleted under these latter harsh oxidizing conditions (i.e., a weak peak response eluting at 18.5 min was detected but this response was lower than other eluting forms of oxidized hhMb).

Subsequently, samples of native or HOCl-modified Mb (10 mol HOCl/mol protein) were assessed with mass spectrometry (Figure 4B,C). In the absence of HOCl the vehicle treated hhMb showed a single mass peak with mass 16,951.2 *m*/*z* in the deconvoluted spectrum, which closely matches the previously reported value for native hhMb (16,951.5 *m*/*z*) [28]. Clearly, treatment of hhMb with reagent HOCl yielded a complex mixture of products with the corresponding deconvoluted mass spectrum showing multiple mass peaks, with the major peak responses differing by multiples of ~16 (compare the multiple peaks marked (i–v), Figure 4C with native hhMb sample exposed to PBS as a vehicle control, Figure 4B as listed in the legend to the figure). These mass changes were consistent with successive covalent addition of molecular oxygen to the protein that was previously ascribed to methionine (Met) residue sulfoxidation at Met55 and Met131 and tryptophan (Trp) residue oxidation at Trp 7 and Trp 14 in the hhMb sequence as defined previously in hhMb using LC/MS tandem MS/MS [21]; the extent of amino acid oxidation followed the reactivity order Met55 > Met131 > Trp7 > Trp14 [21]. While addition of molecular oxygen to methionine simply yields the corresponding sulfoxide, oxidation of Trp is more complex with 3-hydroytryptophan forming an equilibrium with the corresponding 3-keto analogue that can subsequently yield kynurenine in the presence of excess oxidant through the intermediate N-formyl kynurenine [29].

Taken together the post-translational modifications identified by mass analyses have collectively revealed that HOCl-mediated hhMb oxidation yielded a mixture of chlorinated protein (relatively minor product) and a modified protein that contains sequential addition of molecular oxygen (relatively major products) with the extent of oxidation being dependent on the ratio of oxidizing HOCl to target protein employed. Interestingly, we have shown previously that oxidized myoglobin is present in the damaged myocardium after experimental heart attack in rats, however, no evidence of 3-chlorotyrosine was detected in gel purified rat heart Mb [30], which is completely consistent with the majority of HOCl oxidation of Mb yielding serial addition of molecular oxygen at redox active amino acids rather than producing detectable tyrosine chlorination.

**Figure 5:**
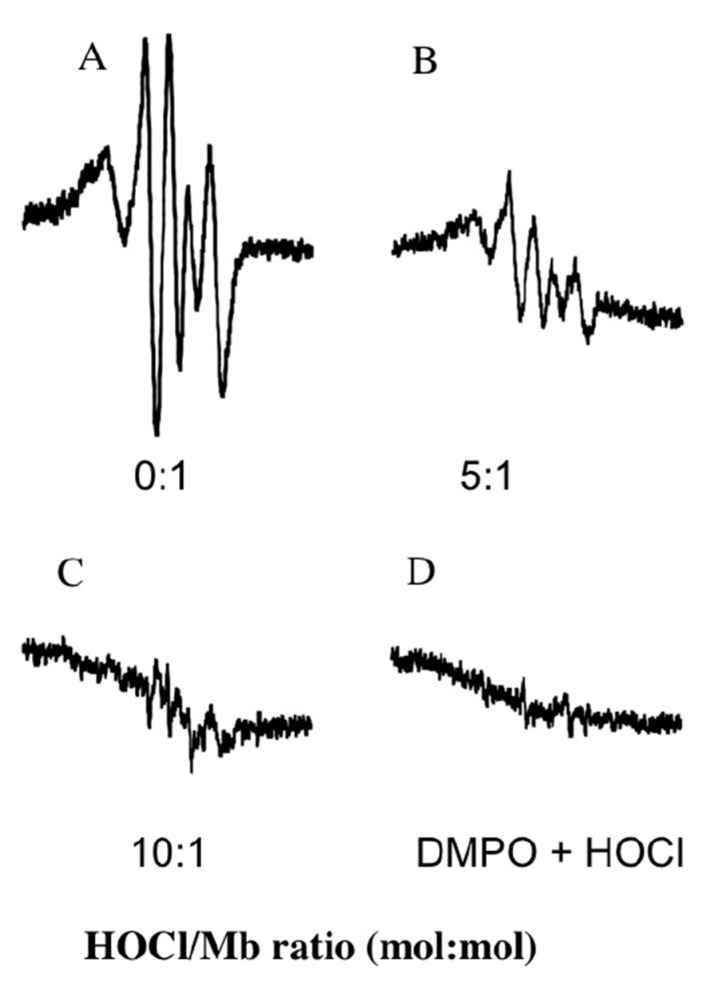
Treatment of hhMb with regent HOCl inhibits the formation of protein-centered radicals as detected by spin trapping. Solutions of hhMb (final concentration 500 µM) were exposed to an increasing dose of reagent HOCl (final oxidant:protein mol ratio indicated in the representative spectra shown in panels (**A**) Control; (**B**) hhMb pretreated with HOCl 5 mol/mol protein; (**C**) hhMb pretreated with HOCl 10 mol/mol protein and (**D**) absence of hhMb). All hhMb solutions contained 100 µM the chelator diethylenetriaminepentaacetic acid (DTPA). Aliquots of reaction mixture were then treated with hydrogen peroxide (final ratio H_2_O_2_:hhMb ∼5 mol/mol) or phosphate buffer (as a vehicle control) in the presence of 5 mM 5,5-dimethyl-1-pyrroline N-oxide (DMPO) as described in the Methods section. The reaction mixture was then rapidly transferred to a flat cell and radical generation assessed using EPR spectroscopy with the following parameters: Microwave power 20 mW, modulation amplitude 0.1 mT, modulation frequency of 100 kHz and a sweep time of 84 s. EPR spectra were obtained as a cumulative average of five consecutive scans. No signal was obtained in the absence of DMPO or H_2_O_2_ (data not shown). Data are representative of *n* = 3 independent experiments using freshly prepared protein radicals.

Under physiological conditions myoglobin reacts with H_2_O_2_ to neutralize the oxidants in a peroxidase-like reaction that yields water and a protein radical (designated as a myoglobin ferryl porphyrin cation radical (MbFe(IV)=O^+^^•^)) that can be translocated through an intramolecular electron-transfer reaction with various reactive amino acids on the protein backbone [31,32]. As anticipated, the reaction of native hhMb with H_2_O_2_ at 22 °C yielded a DMPO spin-trapped adduct, in the absence of reagent HOCl, with a characteristic hyperfine coupling pattern that has been previously assigned to a tyrosyl radical generated at tyrosine residue 103, the Tyr residue residing closest to the Mb-haem prosthetic group [31]. Overall, pre-treatment of hhMb with reagent HOCl dose-dependently decreased the level of spin-trapped tyrosyl radical detected by EPR spectroscopy (Figure 5A–C). By contrast, in the absence of hhMb no significant signal was detected (Figure 5D). Taken together this outcome can be interpreted in two possible ways (i) HOCl-mediated modification of the protein decreases Mb pseudo-peroxidase activity or (ii) HOCl-mediated modification of the protein has no impact on the generation of MbFe(IV)=O^+^^•^ but instead inhibits the intramolecular electron translocation of the MbFe(IV)=O^+^^•^ to the protein backbone, a process which is documented to involve either intramolecular reduction of the MbFe(IV)=O^+^^•^ by amino acids such as tryptophan and tyrosine within hhMb [22,33]; or the intermolecular reaction with tyrosine residues on other proteins [34].

**Figure 6:**
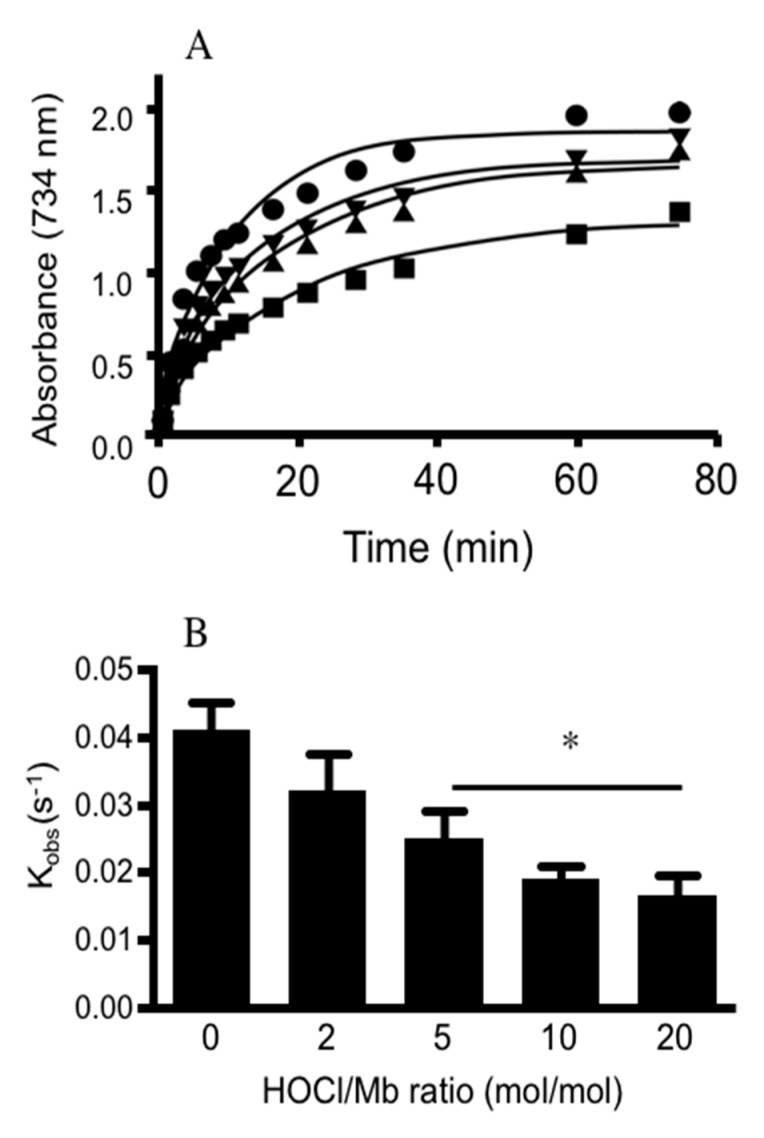
Treatment of hhMb with regent HOCl inhibits ABTS oxidation initiated by H_2_O_2_. Solutions of hhMb containing the chelator DTPA (100 µM) were exposed to vehicle (control) or increasing dose of reagent HOCl (final ratio hhMb/HOCl mol/mol as indicated). Aliquots of control or HOCl-modified hhMb were then transferred to a quartz cuvette containing excess ABTS and hydrogen peroxide (final ratio H_2_O_2_:hhMb ∼5 mol/mol) or excess ABTS with phosphate buffer (50 mM, pH 7.4, control) as described in the Methods section. For all studies the final hhMb concentration in the cuvette was maintained at 25 µM. Next, (**A**) the rate of ABTS radical cation formation was monitored over time at 734 nm. Reaction conditions were as follows: (closed circle) vehicle control; (inverted triangle) hhMb treated with 2 mol/mol HOCl; (filled triangle) hhMb treated with 5 mol/mol HOCl and (filled square) hhMb treated with 10 mol/mol HOCl. Note for clarity samples treated with 20 mol/mol HOCl were not shown on the graph. (**B**) First order rate constants were then determined from the initial rapid increase in ABTS oxidation and plotted against HOCl/hhMb ratio in the reaction mixture. Data represent mean ± SD; *n* = 3 independent kinetic studies using different preparations of hhMb. *Different to the vehicle control in the absence of added reagent HOCl; *p* < 0.05.

To explore whether Mb pseudo-peroxidase activity was altered, we assessed the ability for hhMb to oxidize ABTS and compared rates of ABTS oxidation in the presence of native and HOCl-modified hhMb. Overall, the pseudo-first order rate constant for ABTS oxidation by mixtures of hhMb/H_2_O_2_ decreased in a HOCl-dose dependent manner (Figure 6) indicating that post-translational modification of hhMb affected its ability to oxidize an exogenous substrate (ABTS). This outcome, identified here for the first time, suggests that the reaction between Fe(III)Mb and peroxide to form MbFe(IV)=O^+^^•^ (that is subsequently reduced by the exogenous substrate ABTS) is less efficient when hhMb is first oxidized with HOCl. Therefore, in reactions between H_2_O_2_ and HOCl-modified hhMb the capacity to generate the porphyrin radical cation is inhibited and therefore, it is likely that fewer protein radicals are formed by intramolecular electron transfer between the porphyrin radical cation to the protein backbone simply due to a decreased availability of the MbFe(IV)=O^+^^•^ as a result of the diminished peroxidase activity for HOCl-modified hhMb.

**Figure 7:**
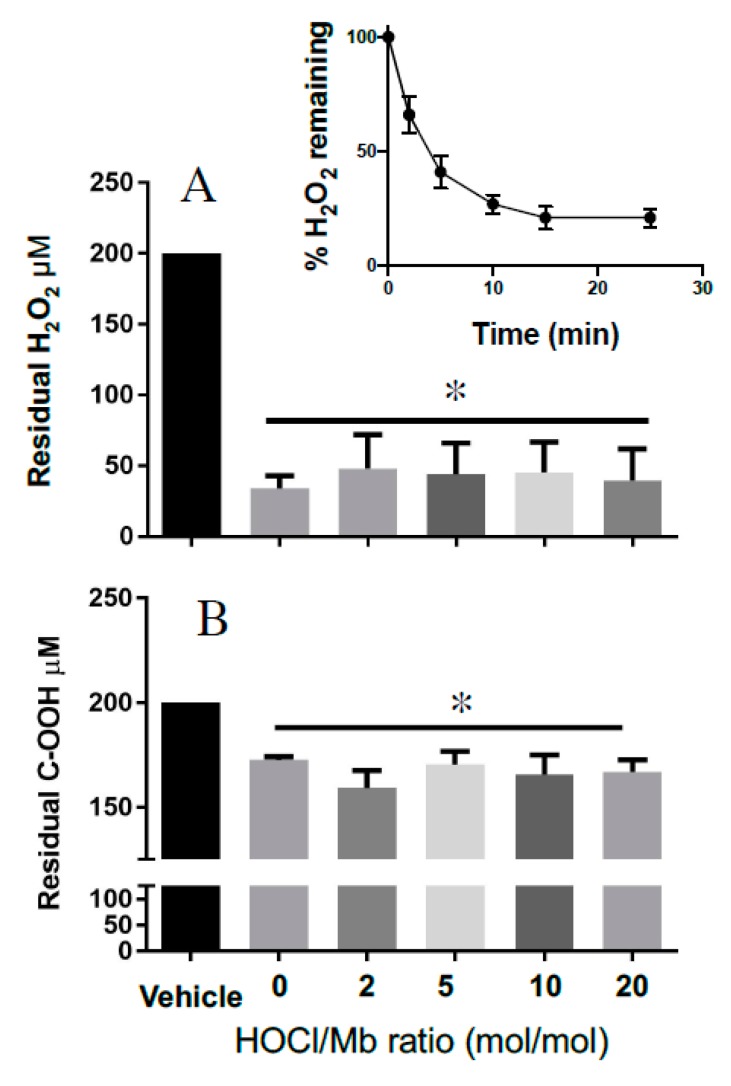
Catalase-like activity of hhMb is unaltered by oxidation with reagent HOCl. Samples of native or reagent HOCl-oxidized hhMb (HOCl:Mb ratio as indicated in the figure; final concentration of hhMb 25 μM) were added to a reaction mixture containing ~0.2 mM peroxide for 15 min, then the mixture was treated with xylenol orange and residual peroxide calculated using a modified FOX-1 assay as described in detail in the Methods section. After mixing in a quartz cuvette the reaction was placed in a spectrometer and peak absorbance was monitored at 560 nm at 22 °C. Panels show Mb-mediated degradation of (**A**) H_2_O_2_ and (**B**) cumene hydroperoxide (C-OOH). Data represent the mean ± SD; *n* = 3 independent experiments using different batches of native hhMb and freshly prepared HOCl-modified hhMb. * Different to the vehicle control in the absence of added reagent HOCl; *p* < 0.05.

Next, we evaluated the impact of reagent HOCl on Mb catalase-like activity. This catalytic activity also involves conversion of MbFe(III) to MbFe(IV)=O^+^^•^. However, in the absence of a suitable substrate MbFe(IV)=O^+^^•^ can act as a two-electron oxidant toward excess peroxide, generating water, oxygen and subsequently recycling MbFe(III) in this reaction. Initially, we tested the time-dependent consumption of H_2_O_2_ in the presence of hhMb to ascertain an appropriate time to assess residual peroxide levels after the reaction had reached near completion (i.e., achieved a steady state). Addition of hhMb to a fixed concentration of H_2_O_2_ (200 µM) resulted in a rapid depletion of the peroxide in the first 2–5 min of the reaction; the reaction rate decreased over the ensuing 20 min and achieved a steady-state at ~15 min (Figure 7A, inset); this time was judged as suitable to determine whether HOCl-oxidation of hhMb affected the protein’s catalase-like activity. In contrast with the measurement of peroxidase activity that was sensitive to reagent HOCl, steady state determinations of hhMb catalase-like activity remained unaffected by HOCl as judged by the similar extent of H_2_O_2_ degradation (consistently ~80–85% of the initial H_2_O_2_ concentration was consumed) in the presence of native or HOCl-oxidized hhMb under the same experimental conditions (Figure 7A). That is, while the depletion of H_2_O_2_ was significantly different for all the hhMb proteins compared with vehicle alone (addition of vehicle containing no protein) there was no difference in the extent of H_2_O_2_ depletion amongst the native or HOCl-oxidized proteins tested. This ability for hhMb to act as a catalase antioxidant mimetic was relatively selective for H_2_O_2_ since degradation of an organic peroxide (cumene hydroperoxide; C–OOH) was markedly less efficient than the degradation of H_2_O_2_ under the identical reaction conditions (comparatively only ~12–18% of the initial C–OOH concentration is consumed; c.f., Figure 7A,B). Interestingly, this diminished capacity to degrade the organic peroxide-OOH also remained independent of the extent of HOCl oxidation as the extent of C–OOH degradation remained largely the same for both native and all HOCl-modified hhMb tested. That is, similar to the case with H_2_O_2_, the depletion of C–OOH was significantly different for all the hhMb proteins compared with vehicle alone (compared with vehicle-treated peroxide containing no protein) yet there was no difference in the extent of cumene hydroperoxide depletion when comparing the native and HOCl-oxidized proteins tested.

**Figure 8:**
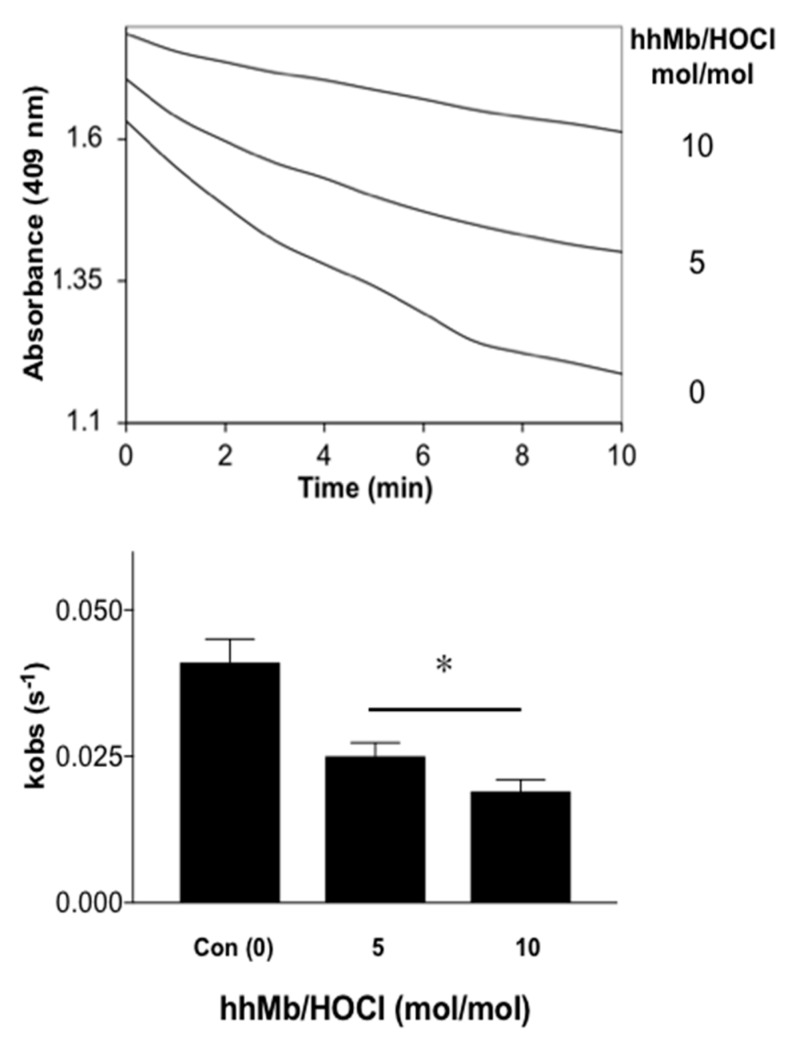
Rates of reduction of Fe(III)-to-Fe(II) in native and HOCl-modified hhMb by a cytochrome *b*5 reductase system. Samples of native or reagent HOCl-oxidized hhMb (final HOCl:Mb ratio 5 or 10 mol oxidant:mol protein; final concentration of hhMb 10 μM) were added to a reaction mixture containing ~0.2 μM recombinant cytochrome *b*5 protein, 0.1 μM NADPH-P450 reductase and an enzymic reductase system as described in detail in the Methods section. After mixing in a quartz cuvette the reaction was placed in a spectrometer and peak absorbance was monitored at 409 nm and 25 °C. (**A**) Time-dependent change in A_409 nm_ for cytochrome *b*5-mediated one-electron reduction of hhMb. (**B**) First-order rate constant estimated for the kinetic reduction of hhMb over the initial linear phase (0–10 min). Data represent the mean ± SD; *n* = 3 independent experiments using different batches of Mb and fresh solutions of cytochrome *b*5 and reductase system. * Different to the vehicle control in the absence of added reagent HOCl; *p* < 0.05.

Finally, to test whether HOCl-induced modifications in hhMb impacted the rate or extent of one-electron reduction by a reduced cytochrome *b*5 reductase system we monitored changes in the rate constant of intermolecular electron transfer to the haem-iron bound within hhMb through monitoring the Soret region with UV-Vis spectroscopy. Overall, similar to the case for recombinant human Mb [35], pre-treatment of hhMb with reagent HOCl decreased the cytochrome *b*5-mediated reduction of hhMbFe(III) haem in a dose-dependent manner and resulted in a significantly decreased observed rate constant for hhMb one-electron reduction compared to native (unmodified) hhMb measured under identical experimental conditions (Figure 8). As maintenance of the MbFe(II) form is essential for oxygen binding and transport in muscle tissues, any change in the rate of chemical reduction of the haem-iron centre in hhMb may impact on the capacity of the protein to perform this function and subsequently impact on tissue oxygenation levels and oxygen delivery to mitochondria.

## 4. Discussion

Exposure to HOCl causes changes in myoglobin structure as judged by a series of biochemical outcomes including changes to electrophoretic mobility and absorbance in the visible region of the hhMb electronic spectrum together with the confirmation that HOCl-oxidized hhMb contains both oxygenated (major) and chlorinated (minor) products; albeit that chlorination is a minor pathway compared with a HOCl-dose dependent serial addition of molecular oxygen to Mb residues Met and Trp. These post-translational modifications resulted in an altered capacity to react with H_2_O_2_ (that is, a diminished pseudo-peroxidase activity) and inhibited the rate of intermolecular one-electron reduction between hhMbFe(III) and a cytochrome *b*5 reductase system [15], although oxidation by reagent HOCl had no material bearing on Mb catalase-like antioxidant activity. Of biological consequence, it is established that Mb exists in high abundance in the myocardium [7] and is an essential oxygen binding protein. Therefore, alterations to its structure and function through oxidative modification under acute inflammatory conditions has the potential to negatively impact on Mb-mediated oxygen storage and transport rendering cardiac muscle vulnerable to hypoxia-reoxygenation injury. If this were to occur in the inflamed myocardium, where recruitment of neutrophils to the periphery of the infarct occurs post AMI, then cardiomyocytes surrounding the primary infarct region may undergo secondary oxidant stress from HOCl produced by the infiltrating neutrophils leading to an expansion of the necrotic region that further diminishes ventricular function.

Data generated here demonstrates that HOCl-mediated oxidation of hhMb impacted Mb pseudo-peroxidase activity yielding a HOCl-dose-dependent inhibition of ABTS oxidation coupled with a diminished capacity to produce protein-centered radicals (specifically tyrosyl radicals localized to Tyr103) in hhMb [36]. Formation of relatively long-lived protein tyrosyl radicals are a hallmark of the reaction between mammalian Mbs and H_2_O_2_ [22,31,37] and subsequent covalent crosslinking of the haem prosthetic group to the protein reportedly involves Tyr103 [38]. However, exposure of hhMb to reagent HOCl inhibits the translocation of oxidizing equivalence to the protein in the presence of H_2_O_2_ suggesting that intramolecular electron transfer from redox active amino acids within the Mb protein to the MbFe(IV)=O^+^^•^ radical (referred to as Mb-compound I) has been impeded, which should decrease intramolecular secondary reduction reactions involving redox active amino acids and paradoxically enhance peroxidase activity through cycling Mb-compound I to the ferryl-iron(IV)-oxo haem species (MbFe(IV)=O; referred to as Mb-compound II), which then cycles further to the resting state MbFe(III) ready for peroxidase activation by excess H_2_O_2_.

Through identifying specific amino acids that are modified by HOCl in mammalian Mbs we have developed an understanding of the order of damage to amino acids in the protein sequence [21] with the order of susceptibility to HOCl-mediated oxidation according to the sequence Met55 > Met131 > Trp7 > Trp14. Interestingly, Trp radicals are detected using low temperature electron paramagnetic spectroscopy techniques [32] and are formed prior to tyrosyl radicals via intramolecular electron transfer reactions involving Mb compound I and Trp7/14; subsequently a second intramolecular electron transfer event yields tyrosyl radicals as the radical sink in mammalian Mbs [31,36]. Therefore, it is feasible that HOCl-mediated Trp oxidation inhibits the downstream formation of Trp protein radicals and this leads to the diminished detection of DMPO-trapped Tyr radicals (Figure 5).

Both Mb compound I and compound II can oxidize substrates in the peroxidase cycle, which is the case for professional peroxidase enzymes such as horse radish peroxidase that are able to cycle at high turnover in the absence of self-oxidation [39,40]. The precise mechanism that explains this decreased peroxidase activity through inhibiting electron transfer processes between the haem-iron center and amino acid residues within the protein were not explored completely in this study, albeit that diminished production of Mb compound I was implied by our data. However, it is clear that tyrosine chlorination is unlikely to be a major pathway that impedes peroxidase enzyme turnover since the absolute proportion of 3-Cl-Tyr/native tyrosine was extremely low in HOCl-oxidized hhMb (in the order 2.4 chlorination products per 1000 Tyr residues), indicating that a majority of tyrosine in the protein was not modified by reagent HOCl and therefore, this amino acid remained available to reduce MbFe(IV)=O^+^^•^ that formed in reaction with peroxide. However, spin trapping of the hhMb-tyrosyl radical with DMPO indicated that formation of the tyrosine phenoxyl radicals was diminished by HOCl in a dose-dependent fashion hence we concluded that HOCl-modification of hhMb impacts the formation of Mb compound I. In contrast to Tyr, HOCl-oxidized damage to other amino acids such as Trp and Met predominate in Mb products [21]. Thus, it is possible that Trp and Met oxidation also play a more significant role in altering Mb peroxidase activity, possibly through alterations in protein tertiary structure, and this may have pathophysiological implications considering that at least these same methionine oxidation products are detected in the myocardium following experimental heart attack [30]. Other possibilities include oxidation of amino acid residues that impact on protein structure causing (i) altered haem-iron reactivity to added H_2_O_2_ due to changes in the hydrogen bonding network within the active site cavity or (ii) changes to the redox activity of the haem-iron centre as a result of altered interactions between the haem prosthetic group yielding changes to conformational binding of the iron atom that impact the redox potential for the Fe-centre. Notably, distal-H bonding effects between amino acids within the protein and the haem-centre in the active site are known to regulate haem-iron redox characteristics and ligand binding [41] and previous studies using site-directed mutation of histidine residues in the active site are known to alter Mb peroxidase activity [42]. Therefore, despite no direct evidence for altered histidine in HOCl-oxidized hhMb, oxidative modifications to other redox sensitive amino acids in hhMb may well be responsible for changing distal-H bonding effects in the protein as manifested here by the altered electronic spectral features for HOCl-modified hhMb [43] and decreased peroxidase activity demonstrated by the inhibition of ABTS oxidation (Figure 6).

The catalase-like antioxidant activity of haem peroxidase proteins is known to occur through a mechanism that involves the intermediates that are formed when a professional peroxidase enzyme reacts with H_2_O_2_ [14]. This catalase mimetic activity is further limited by the presence of a suitable electron-donating substrate as this promotes the peroxidase pathway in favor of H_2_O_2_ degradation. In the absence of a suitable reductant Mb-mediated H_2_O_2_ degradation involves formation of MbFe(IV)=O^+^^•^/Mb compound I (Equation (1)) and a subsequent oxidation reaction between MbFe(IV)=O^+^^•^/Mb compound I and peroxide that recycles MbFe(III) and yields water and molecular oxygen (Equation (2)); this is the rate limiting step in this catalase-like activity [44]. Interestingly, point mutations of histidine 93 in human Mb [45] or histidine 64 in other mammalian Mb [46] are known to stabilize MbFe(IV)=O^+^^•^ and enhance Mb catalase-like antioxidant activity.

MbFe(III) + H_2_O_2_ → MbFe(IV) = O^+^^•^ + H_2_O (1)

MbFe(IV) = O^+^^•^ + H_2_O_2_ → MbFe(III) + H_2_O + O_2_ (2)

By comparison our data likely indicate that stabilization of MbFe(IV)=O^+^^•^ in HOCl-oxidized hhMb (through a mechanism involving inhibited intra-molecular translocation of the radical cation from MbFe(IV)=O^+^^•^/Mb compound I to the protein backbone through chemical reduction by redox active amino acids; Figure 5) has no meaningful effect on Mb catalase-like antioxidant activity with the extent of peroxide degradation being the same for both native (in the absence of HOCl-treatment) and HOCl-modified hhMb (up to HOCl/hhMb = 20 mol/mol; Figure 6A). Maintenance of the catalase-like antioxidant activity is contrasted by a marked decrease in pseudoperoxidase activity of HOCl-modified hhMb, which indicates that reaction of MbFe(IV)=O^+^^•^ and the exogenous substrate ABTS occurs with a lower rate constant, possibly reflecting a lower absolute concentration of MbFe(IV)=O^+^^•^ as a consequence of HOCl-oxidation of the protein. Overall, the high concentration of Mb in cardiac tissues suggests that this haem protein may complement endogenous catalase in degrading excessive H_2_O_2_, particularly during AMI where hypoxic insult coupled with reoxygenation injury results in acute peroxide production. Therefore, the ability for HOCl-oxidized Mb to maintain catalase-like antioxidant activity may allow cardiac myoglobin to continue to degrade accumulating H_2_O_2_ under conditions where HOCl are also produced in high concentration post AMI, thereby inhibiting ongoing oxidative stress in the affected myocardium upon resumption of blood flow (and thereby, limiting reperfusion injury).

Exposure of ferric hhMb to regent HOCl alters the ferric-to-ferrous haem reduction by a cytochrome *b*5 reductase system [15], which is considered the most likely intercellular reductase system responsible for maintaining the ferrous Mb form suitable for intracellular oxygen binding and storage [26]. Previously we reported that reagent HOCl oxidized several residues in human myoglobin resulting in decreased rates of MbFe(III) reduction with cytochrome *b*5 reductase [35] and herein we recapitulate this outcome with hhMb and demonstrate a similar reagent HOCl dose-dependent decrease in the rate of one-electron reduction of ferric hhMb by the MetMb reductase system. As such, interruption of ferric-to-ferrous recycling decreases the potential for Mb to continue efficient oxygen transport to mitochondria [47] since MbFe(III) is unable to bind to molecular oxygen. Myoglobin is essential for oxygen supply [48] therefore decreased ATP production as a result of decreased oxygen supply, disrupts the active Na^+^-K^+^-ATPase pump leading to alterations in the level of sodium ions [49], which in turn stimulates the release of calcium ions through the activity of the membrane sodium-calcium exchanger transporter. Excessive calcium levels further damage mitochondria and render the cell more susceptible to oxidative damage and apoptosis, which are all hall marks of the necrotic myocardium post-AMI and other pathologies where Mb is implicated such as rhabdomyolysis-mediated renal damage [50].

Mammalian Mb also displays other functions such as nitrate reductase [51] and lipid metabolism activities [52]. Whether HOCl modification of Mb impacts these other functions is not clear. However, if found to be true this would suggest that HOCl-mediated changes to Mb function may have wide ranging pathophysiological impact, especially in tissues where Mb is found in relatively high concentration, such as the heart. For example, under physiological conditions, Mb acts as a NO scavenger thereby limiting its damaging effect on mitochondrial respiration however in ischemic conditions such as post myocardial infarction, Mb is capable of generating NO with the potential to promote vasodilation of blood vessels thereby increasing oxygen supply to affected myocytes [53]. Furthermore, increased NO production in the myocardium after AMI inhibits mitochondrial complex I known to generate reactive oxygen species post reperfusion. Notably, NO also inhibits the opening of the mitochondrial permeability transition pore involved in cytochrome *c* release and apoptosis post reperfusion [54]. Thus, functional Mb may be critical to the recovery of the damaged myocardium post-AMI by acting through multiple pathways involving different activities where oxygen binding to MbFe(II) is central to maintenance of those activities. Through altering Mb function, neutrophil-HOCl may then promote further damage to the myocardium following AMI and this may be linked to longer-term cardiac disorders such as heart failure.

## 5. Conclusions

The present study has documented that reagent HOCl is capable of promoting post-translational changes to hhMb that impact selected biochemical activities of the protein. The post-translational changes detected in hhMb (here) closely mimic post-translational modification previously determined for horse [21] and human [34] Mb indicating that these changes are likely to be common amongst mammalian Mbs with similar protein sequences. Furthermore, as we have previously described for human Mb, HOCl-modified hhMbFe(III) exhibits decreased one-electron reduction to the MbFe(II) form that diminishes the capacity for oxygen binding/transport. This present study expands on this knowledge base and now shows that Mb catalase-like activity is maintained even in the presence of HOCl at an oxidant:protein ratio up to 20 mol/mol. However, this is not the case for Mb pseudo-peroxidase activity, which was demonstrated here to be inhibited by HOCl-mediated post-translational modification. Therefore, Mb catalase-like activity will act in concert with native catalase to neutralize excessive H_2_O_2_ thus minimizing the damage to cardiomyocytes. If peroxide overwhelms the compensatory catalase like activity of Mb then suppression of the peroxidase activity may be important as peroxidase enzymatic cycling can cause oxidation of a range of biological targets including aromatic substrates/antioxidants/redox sensitive thiols thereby impacting the physiological levels of these biomolecules. The potential for HOCl-oxidized Mb to potentiate cardiac damage under these conditions warrants further investigation in animal models of heart attack where at least some of these post-translationally modified Mb oxidation products have been previously detected [30].
